# Involvement of Workers in Closed and Semiclosed Institutions in Outbreaks of Acute Gastroenteritis Due to Norovirus

**DOI:** 10.3390/v12121392

**Published:** 2020-12-04

**Authors:** Ignacio Parrón, Irene Barrabeig, Miquel Alseda, Thais Cornejo-Sánchez, Susana Guix, Mireia Jané, Conchita Izquierdo, Cristina Rius, Àngela Domínguez, the Working Group for the Study of Acute Gastroenteritis Outbreaks in Catalonia

**Affiliations:** 1 Sub-Direcció Regional a Barcelona del Departament de Salut, 08005 Barcelona, Spain; ibarrabeig@gencat.cat; 2 CIBER Epidemiologia y Salud Pública, Instituto de Salud Carlos III, 28029 Madrid, Spain; mireia.jane@gencat.cat (M.J.); crius@aspb.cat (C.R.); angela.dominguez@ub.edu (À.D.); 3 Sub-Direcció Regional a Lleida del Departament de Salut, 25006 Lleida, Spain; miquel.alseda@gencat.cat; 4 Departament de Microbiologia, Vall d’Hebrón Hospital, 08035 Barcelona, Spain; tcornejo@vhebron.net; 5 Enteric Virus Laboratory, Department of Genetics, Microbiology and Statistics, Section of Microbiology, Virology and Biotechnology, School of Biology, Institute of Nutrition and Food Safety, University of Barcelona, 08028 Barcelona, Spain; susanaguix@ub.edu; 6 Sub-Direcció General de Vigilància i Resposta a Emergències de Salut Pública, 08005 Barcelona, Spain; conchita.izquierdo@gencat.cat; 7 Departament de Medicina, Universitat de Barcelona, 08036 Barcelona, Spain; 8 Agència de Salut Pública de Barcelona, 08023 Barcelona, Spain

**Keywords:** norovirus, outbreak, acute gastroenteritis, closed institution, semiclosed institution, workers

## Abstract

Norovirus outbreaks frequently occur in closed or semiclosed institutions. Recent studies in Catalonia and various countries indicate that, during outbreaks in these institutions, norovirus is detected in between 23% and 60% of workers, and the prevalence of infection in asymptomatic workers involved in outbreaks ranges from 17% to 40%. In this work, we carried out a prospective study to investigate the involvement of workers in closed and semiclosed institutions during outbreaks. The attack rates (ARs) and the rate ratios (RRs) were calculated according to the type of transmission and occupational category. The RRs and 95% confidence intervals (CIs) between workers and users were calculated. The mean cycle of quantification (Cq) values were compared according to the genogroup and the presence of symptoms. ARs were higher in person-to-person transmission than in common vehicle outbreaks, and 38.8% of workers were symptomatic. The RR between workers and users was 0.46 (95% CI 0.41–0.52). The ARs in workers were high, particularly in workers with closer contact with users. The mean Cq was lower in patients than in asymptomatic infected persons, although the difference was only significant for genogroup I (GI). The frequency of asymptomatic infected persons suggests that personal hygiene measures should be followed by all workers in the centers affected.

## 1. Introduction

Most acute gastroenteritis (AGE) cases worldwide are caused by viruses. The causes include, in addition to norovirus, other enteric viruses such as adenovirus, astrovirus, rotavirus and sapovirus, although norovirus is the most common causal agent [1,2,3]. Noroviruses are nonenveloped RNA viruses of the *Caliciviridae* family. There are six genogroups (GI to GVI), although only genogroups I, II and IV are human pathogens [4,5].

The transmission mechanism is fecal–oral, and direct person-to-person contact is a very efficient mechanism. Transmission by food, water and fomites is common, and transmission by aerosols generated by vomiting has been described [6].

A systematic review estimated that human caliciviruses (including genogroup I and II norovirus and sapovirus) caused 71,000 deaths worldwide in children aged <5 years in 2011 [7].

Norovirus is highly resistant to high levels of chlorine [8], heat, cold [9], acidic pH and organic solvents [10,11], which allows its survival for long periods in the environment [12] and facilitates its transmission. The high transmissibility of norovirus is also facilitated by the short duration of immune protection [13], the low infectious dose and the frequency of asymptomatic infections and because the presence of norovirus viruses in feces may be prolonged in both symptomatic and asymptomatic infected persons.

The doses that cause infection in 50% of exposed people (ID_50_) described to date range from 18 to 2934 viral genomes [14,15,16,17]. About 30% of people affected by norovirus have an asymptomatic infection [18].

AGE outbreaks due to norovirus frequently occur in closed or semiclosed institutions such as long-term care facilities, daycare centers, schools, nursing homes and hotels. According to 2006–2010 data, in Catalonia (Spain), >50% of norovirus outbreaks occurred in these types of institutions [19].

The measures to control outbreaks in Catalonia include the recommendation that workers with acute gastroenteritis due to norovirus do not return to work until >48 h after the end of symptoms [20].

Recent studies in Catalonia and various countries indicate that, during outbreaks in these institutions, norovirus in feces is detected in between 23% [21] and 60% [22] of workers and the prevalence of infection in asymptomatic workers involved in outbreaks ranges from 17% [21] to 40% [22].

Although few studies have investigated the involvement of workers in AGE outbreaks due to norovirus in closed and semiclosed institutions, the attack rate among workers may vary depending on the demographic characteristics and type of occupation, the type of institution and the type of transmission causing the outbreak.

The objective of the study was to investigate the involvement of workers in outbreaks due to norovirus in closed and semiclosed institutions according to type of center, type of transmission, genogroups involved and viral load.

## 2. Materials and Methods

### 2.1. Type of Study, Study Period and Study Population

This was a prospective study of AGE outbreaks due to norovirus reported in 2017–2019 to the Notifiable Diseases System of Catalonia [23], a region in the northeast of Spain with a population of 7,496,276 in January 2017 [24].

### 2.2. Outbreaks Included in the Study

All laboratory-confirmed AGE outbreaks due to norovirus that occurred in closed and semiclosed institutions during the study period were included.

A closed institution was defined as one in which users remained the whole day, including the night, although they might leave for short periods for exceptional reasons. Within this category, we included nursing homes, long-term care facilities and summer camps, among others. A semiclosed institution was defined as one carrying out social or educational activities (nonoccupational) in which users remained for >8 h per day, and in which most users consumed at least one meal. Daycare centers, preschool centers, schools and hotels were included in this category.

AGE was defined as sudden-onset diarrhea accompanied by nausea, vomiting, abdominal pain or fever. A norovirus outbreak was defined as AGE in ≥2 people with a common vehicle or person-to-person transmission with norovirus in stool samples identified by real-time reverse transcription polymerase chain reaction (RTqPCR) [25,26].

### 2.3. Data Collection

Data on the outbreaks included were collected by technicians of the Epidemiological Surveillance Services of the Public Health Agency of Catalonia and the Public Health Agency of Barcelona.

In all reported outbreaks, the type of institution, the number of users, the number of workers and the type of transmission (person-to-person or common vehicle) were recorded.

All persons exposed were questioned about sociodemographic variables (sex and date of birth) and their relationship with the institution (worker or user). Information on the type of occupation and the presence of clinical symptomatology was also collected in workers.

Samples of feces were collected from workers and users to identify norovirus genogroups I, II and IV by RTqPCR. Samples were analyzed in the Microbiology Laboratory of Vall d’Hebron University Hospital. The specific primers described by Kageyama et al. were used to detect norovirus GI and GII [25]. A modification of the primer described by Farkas et al. [26] and Kageyama et al. [25] was used to detect norovirus GIV.

### 2.4. Data Analysis and Management

The proportions of the study variables and their 95% confidence intervals (CIs) were calculated. The global attack rates, the attack rates and the rate ratios (RRs) and their 95% CIs were calculated for the mode of transmission (person-to-person or common vehicle), type of institution and type of work activity.

To estimate the risk of workers becoming ill with respect to users, the RRs and their 95% CIs were calculated.

To estimate the viral load, the mean cycle of quantification (Cq) values obtained by RTqPCR [27] were calculated. The means of Cq were compared using the Student’s *t*-test. Statistical significance was established as *p* < 0.05. Data were collected and handled using Microsoft Access 12.0 database manager, and the PASW Statistics 18.0.2 statistical package was used for the statistical analysis.

## 3. Results

**Figure 1:**
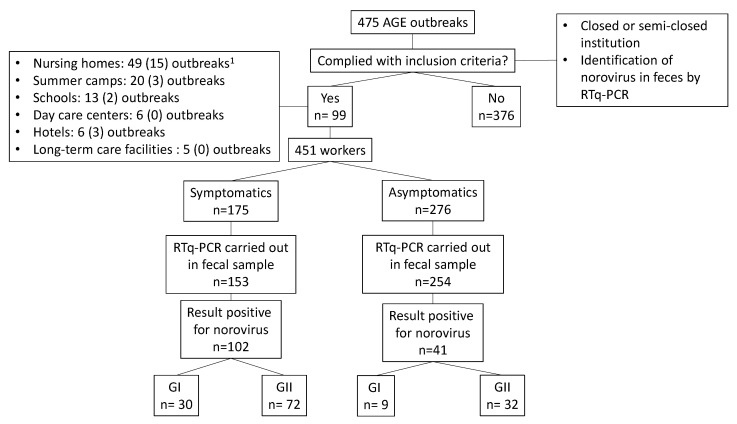
Study flowchart. ^1^ In brackets, the number of outbreaks in which the first case was a worker.

During the study period, 99 AGE outbreaks due to norovirus were detected in closed or semiclosed institutions (26 in 2017, 33 in 2018 and 40 in 2019): 49 in nursing homes (49.5%), 20 in summer camps (22.2%), 13 in schools (13.1%), 6 in daycare centers (6.1%), 6 in hotels (6.1%) and 5 in long-term care facilities (5.1%) (Figure 1).

In 74 outbreaks (74.8%), transmission was person-to-person; in the remaining 25 (25.2%), it was due to a common vehicle.

The epidemiological survey was answered by 451 workers and 1015 users from the affected centers. Of the workers, 175 (38.8%) were symptomatic and 276 (61.2%) had no symptoms.

The attack rate was 32.03% in males and 41.49% in females. No significant differences in attack rates were found according to sex or age group (Table 1).

The attack rate in workers was 43.9% in person-to-person outbreaks and 32.6% in outbreaks with a common vehicle (Table 2). The risk of workers being symptomatic was higher in person-to-person outbreaks than in those with a common vehicle (RR 1.35; 95% CI 1.05–1.74). Analysis by type of institution showed the RR of attack rates was only significant for schools (RR 1.93; 95% CI 1.07–3.49).

A total of 1015 users responded to the epidemiological survey, of whom 854 were users who were symptomatic (attack rate 84.1%). The RR of attack rates between workers and users was 0.46 (95% CI 0.41–0.52). The lower risk of workers compared with users was also observed separately for each type of institution (Table 3).

The attack rates differed according to the type of institution; the highest rates were for long-term care facilities (86.15%) and nursing homes (75.73%), and the lowest rates were for hotels (34.83%). Globally, in all types of institutions, workers had significantly lower attack rates than users.

With respect to the type of occupation, caregivers in nursing homes and healthcare workers had an increased risk of becoming ill, while being a kitchen worker was a protective factor against infection (Table 4).

**Figure 2:**
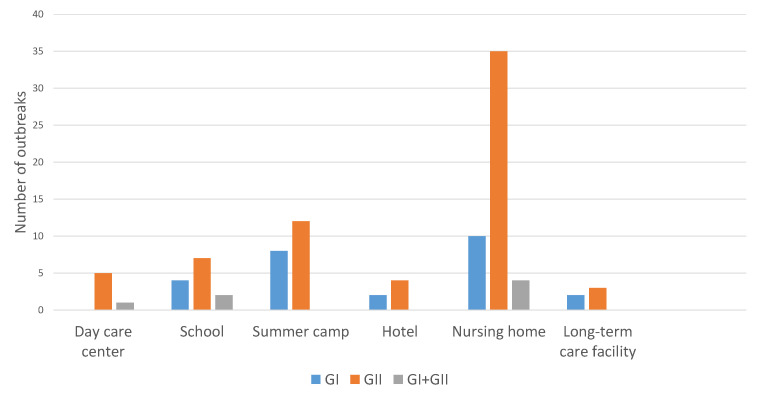
Outbreaks according to type of center and genogroup.

The most common genogroup was norovirus GII with 66 outbreaks (66.7%); 26 were due to GI (26.3%), and the etiology was mixed in 7 (7.1%). No outbreak due to GIV was detected. GII was more frequently involved in all types of institutions than GI (Figure 2).

Norovirus was detected by RTqPCR in 143 workers (102 symptomatic and 41 asymptomatic) and 687 users (603 symptomatic and 84 asymptomatic). Norovirus GI was detected in 30 symptomatic workers, 9 asymptomatic workers, 144 symptomatic users and 19 asymptomatic users. Norovirus GI was detected in 72 symptomatic workers, 32 asymptomatic workers, 459 symptomatic users and 65 asymptomatic users.

The mean viral load, measured indirectly by Cq, was 27.69 for GI and 31.61 for GII, although the values are not comparable as the measurements were made using different tests.

Mean Cq was lower in symptomatic persons than in asymptomatic infected persons, with a higher viral load in symptomatic persons, for genogroups GI and GII. For GI, a mean Cq of 36.97 (SD 5.13) was observed in asymptomatic infected persons and a mean Cq of 30.01 (SD 5.51) was observed in symptomatic persons (*p* = 0.002). Although the Cq was also higher for GII in asymptomatic compared with symptomatic persons (29.19; SD 5.26 vs. 27.01; SD 5.84), the differences were not statistically significant (*p* = 0.07) (Table 5).

## 4. Discussion

The attack rate in workers in institutions where outbreaks included in the study occurred was 38.8%, higher than the 10.45% found by Wu et al. in workers involved in norovirus outbreaks in Shanghai between 2015 and 2017 [21] and the 30% described by Sabria et al. in food handlers and healthcare workers in outbreaks in Catalonia between 2010 and 2012 [22]. The higher attack found in our study may be because the studies mentioned were not limited to closed or semiclosed institutions, in which transmission occurs more easily than in other types of institutions.

We found that 16.14% of asymptomatic workers in AGE outbreaks due to norovirus in closed and semiclosed institutions were infected.

Asymptomatic norovirus infection is common, even among people without known exposure. Qi et al., in a meta-analysis of published studies on asymptomatic norovirus infection, found a prevalence of infection of 7% worldwide in the general population [28]. Yu et al. found that 3.3% of food handlers unrelated to outbreaks were asymptomatically infected [29], and Okabayashi et al. found a rate of asymptomatic infections of up to 12% in workers in institutions [30]. 

Wang et al. found norovirus infections in 4.04% of the inhabitants of municipalities related to oyster cultivation, with no differences between workers in oyster farms and the rest of the population [31].

Other studies of workers involved in AGE outbreaks due to norovirus have found very similar rates to those described in our study. Wu et al. found an infection rate of 17% in asymptomatic workers in institutions where outbreaks occurred [21], a rate very similar to ours. Qi et al. found the prevalence of asymptomatic infected people to be 18% in workers related to outbreaks [28]. Our results, in common with other reports [32], found no significant differences in attack rates between male and female workers.

The main transmission route of norovirus is direct person-to-person contact, and its dissemination is facilitated by the conditions in which the outbreak occurs. 

Increased personal contact between individuals, such as in nursing homes, schools and daycare centers, is likely to facilitate greater transmission [33,34]. Godoy et al. found an increased risk in workers in an AGE outbreak due to norovirus in a nursing home when workers had more direct contact with residents [35].

Our results showed the greatest risk of transmission was direct person-to-person transmission rather than transmission by a common vehicle (RR 1.35 95%; CI 1.05 to 1.74) and that the greatest risk was in caregivers in nursing homes and healthcare workers, whose occupational activity involves closer and longer-lasting contact with users. In contrast, kitchen workers, who have less direct contact with users, had a lower risk of being symptomatic.

GII was the most frequently identified genogroup, both in symptomatic and asymptomatic infected persons. The predominance of the GII genogroup, both in isolated cases of AGE and in outbreaks or asymptomatic infections, has been described by various authors. Yu et al. identified GII in 65% of food handlers in elementary schools in the Incheon region (Korea) [29]. Park et al. identified GII in 75% of positive samples from workers in nursing homes with norovirus outbreaks [36].

Likewise, 90.5% of asymptomatic children studied by Qi et al. in a nursery in Changzhou, China, were infected by genogroup GII [37], and in the United States between 2009 and 2015, 81% of norovirus outbreaks were caused by GII [38].

The viral load was higher in symptomatic persons than in asymptomatic infected persons in all cases. While differences in the mean Cq of symptomatic persons vs. asymptomatic infected persons were statistically significant for GI (*p* = 0.002), no significant differences were found for GII (*p* = 0.073).

Teunis et al. found no differences in the viral load between symptomatic persons and asymptomatic infected persons in workers and users of nursing homes and hospitals involved in GII outbreaks [39]. Kabue et al. found that, in children in rural South Africa, the viral load of GII was higher in symptomatic than in asymptomatic persons, but there were no significant differences for GI [40].

The interpretation of the significance of these differences is difficult, given the discrepancies in the results obtained in different studies, but in all cases, viral loads were detected in asymptomatic infected persons, indicating the potential of these people to act as sources of contagion during outbreaks.

The study has some limitations. Firstly, 12.6% of symptomatic workers and 8% of asymptomatic workers were not analyzed using RTqPCR, which could bias the results. However, the differences between these percentages were not significant (*p* = 0.11), and therefore the comparisons between the two categories of infected persons are valid. 

Secondly, the fact that in some studies significant differences in relation to an increased viral load in symptomatic versus asymptomatic persons were observed for genogroup GII compared with genogroup GI may be explained by the sample size or confounding factors, and this could be the subject of further studies. 

Thirdly, our study was carried out using the surveys answered, and it may be that people who became ill were more willing to collaborate in responding to the survey than those who did not present symptoms, which could have resulted in an overestimate of the attack rates.

Fourthly, some data were not available, such as the theoretical total capacity of the affected centers, the density of occupancy at the time of the outbreak or the ratio between workers and users, so the possible influence of these factors on the attack rates observed could not be analyzed.

The main strength of our study was that it was carried out in the context of epidemiological surveillance, and therefore the study coverage was universal in the target population.

## 5. Conclusions

The attack rate in workers in closed and semiclosed institutions was high and was related to the type of activity, being higher in workers with closer contact with users.

The frequency of asymptomatic infected persons suggests that in an AGE outbreak due to norovirus, personal hygiene measures should be followed by all workers in the institution where the outbreak occurred.

Although the genogroup I viral load in symptomatic persons was significantly higher than that in asymptomatic infected persons, the viral loads of asymptomatic infected persons were high for both genogroups GI and GII, indicating the potential of these asymptomatic people as a source of infection.

## Figures and Tables

**Table 1:** Attack rates in workers by age and sex.

	Males			Females			
Age (Years)	Symptomatic	Exposed	Attack Rate	Symptomatic	Exposed	Attack Rate	*p-*Value
17–25	10	17	58.82%	29	45	64.44%	0.69
26–35	11	38	28.95%	28	58	48.28%	0.06
36–45	10	27	37.04%	20	55	36.36%	0.95
46–55	5	24	20.83%	31	83	37.35%	0.13
56–65	4	14	28.57%	14	37	37.84%	0.54
NA	1	8	12.50%	12	45	26.67%	0.35
Total	41	128	32.03%	134	323	41.49%	0.06

NA: not available.

**Table 2:** Attack rates and rate ratios (RRs) in workers according to type of institution and type of transmission.

Type of Institution (Total Affected) ^1^	Symptomatic	Exposed	Attack Rate	RR (95% CI)
Summer camp				
Person-to-person (74)	1	11	9.09%	0.17 (0.02 to 1.30)
Common vehicle (105)	12	32	37.5%	1
Mixed transmission (4)	0	8	0%	NC
Total (183)	13	51	25.49%	
School				
Person-to-person (144)	15	56	26.79%	1.93 (1.07 to 3.49)
Common vehicle (24)	11	69	15.94%	1
Total (168)	26	125	20.80%	
Daycare center				
Person-to-person (69)	6	14	42.86%	NC
Common vehicle (0)	0	0	0%	
Total (69)	6	14	42.86%	
Hotel				
Person-to-person (14)	9	40	22.5%	0.93 (0.44 to 1.95)
Common vehicle (48)	5	21	23.81%	1
Total (62)	14	61	22.95%	
Nursing home				
Person-to-person (400)	77	133	57.89%	1.03 (0.78 to 1.38)
Common vehicle (93)	28	50	56.00%	1
Total (493)	105	183	57.37%	
Long-term care facility				
Person-to-person (55)	11	17	64.70%	NC
Common vehicle (0)	0	0	0%	
Total (55)	11	17	64.70%	
Total				
Person-to-person (756)	119	271	43.91%	1.35 (1.05 to 1.74)
Common vehicle (270)	56	172	32.56%	1
Mixed transmission (4)	0	8	0%	NC
Total (1030)	175	451	38.80%	

^1^ The total number of affected persons, including users and workers, is shown in parentheses. NC: Not calculable.

**Table 3:** Attack rates and rate ratios (RRs) in workers and users according to type of institution.

Type of Institution	Symptomatic	Exposed	Attack Rate	RR (95% CI)
Summer camp				0.30 (0. 19 to 0. 48) 1
Workers	13	51	25.49	
Users	170	201	84.58	
Total	183	252	72.62	
Schools				0.25 (0.17 to 0.35) 1
Workers	26	125	20.80	
Users	141	167	84.43	
Total	167	292	57.19	
Daycare center				0.53 (0.28 to 0.97) 1
Workers	6	14	42.86	
Users	62	76	81.58	
Total	68	90	75.56	
Hotel				0.26 (0.16 to 0.42) 1
Workers	14	61	22.95	
Users	48	55	87.27	
Total	62	116	34.83	
Nursing home				0.69 (0.61 to 0.79) 1
Workers	105	183	57.38	
Users	388	468	82.91	
Total	493	651	75. 73	
Long-term care facility				0.69 (0.48 to 0.99) 1
Workers	11	17	64.71	
Users	45	48	93.75	
Total	56	65	86.15	
Total				0.46 (0.41 to 0.52) 1
Workers	175	451	38.80	
Users	854	1015	84.14	
Total	1029	1466	70.19	

**Table 4:** Attack rates and rate ratios (RRs) in workers according to type of occupation.

Type of Occupation	Attack Rate	RR ^1^ (95% CI)
Cook	9.8%	0.26 (0.12.0.56)
Kitchen assistant	15.0%	0.36 (0.23.0.59)
Waiter	37.5%	1.13 (0.45. 2.79)
Dining monitor	27.8%	0.79 (0.54. 1.14)
Caregiver or healthcare worker	71.6%	3.18 (2.32. 4.35)
Global attack rate	38.8%	

^1^ Workers in each type of occupation compared with all other workers.

**Table 5:** Difference in viral load between symptomatic and infected asymptomatic persons according to genogroup.

Genogroup	Symptomatic	N	Mean Cq	SD	*p-*Value
GI	Yes	30	30.01	5.51	0.002
	No	9	36.97	5.13	
GII	Yes	72	27.01	5.84	0.07
	No	32	29.19	5.26	
